# Inhibition of Mitochondrial Complex II by the Anticancer Agent Lonidamine*

**DOI:** 10.1074/jbc.M115.697516

**Published:** 2015-10-31

**Authors:** Lili Guo, Alexander A. Shestov, Andrew J. Worth, Kavindra Nath, David S. Nelson, Dennis B. Leeper, Jerry D. Glickson, Ian A. Blair

**Affiliations:** From the ‡ Penn Superfund Research and Training Program Center, Center of Excellence in Environmental Toxicology, and Department of Systems Pharmacology and Translational Therapeutics and; § Laboratory of Molecular Imaging Department of Radiology, University of Pennsylvania, Philadelphia, Pennsylvania 19104 and; ¶ Department of Radiation Oncology, Thomas Jefferson University, Philadelphia, Pennsylvania 19107

**Keywords:** anticancer drug, cancer therapy, chromatography, drug action, glucose metabolism, glutamate, glutamine, glycolysis, high-performance liquid chromatography (HPLC), mass spectrometry (MS)

## Abstract

The antitumor agent lonidamine (LND; 1-(2,4-dichlorobenzyl)-1*H*-indazole-3-carboxylic acid) is known to interfere with energy-yielding processes in cancer cells. However, the effect of LND on central energy metabolism has never been fully characterized. In this study, we report that a significant amount of succinate is accumulated in LND-treated cells. LND inhibits the formation of fumarate and malate and suppresses succinate-induced respiration of isolated mitochondria. Utilizing biochemical assays, we determined that LND inhibits the succinate-ubiquinone reductase activity of respiratory complex II without fully blocking succinate dehydrogenase activity. LND also induces cellular reactive oxygen species through complex II, which reduced the viability of the DB-1 melanoma cell line. The ability of LND to promote cell death was potentiated by its suppression of the pentose phosphate pathway, which resulted in inhibition of NADPH and glutathione generation. Using stable isotope tracers in combination with isotopologue analysis, we showed that LND increased glutaminolysis but decreased reductive carboxylation of glutamine-derived α-ketoglutarate. Our findings on the previously uncharacterized effects of LND may provide potential combinational therapeutic approaches for targeting cancer metabolism.

## Introduction

Targeting cancer metabolism has received renewed interest as a strategy for anticancer therapy (1–3). Most cancers have altered metabolism and a high demand for nutrients to support their rapid proliferation (4–7). Warburg *et al.* (8) first reported that malignant cells metabolize glucose via aerobic glycolysis, which is often associated with increased glucose uptake and lactate production. Many other metabolic alterations have been discovered in cancer cells, including a high rate of glutamine consumption, enhanced lipid synthesis, and increased pentose phosphate pathway (PPP)^3^ flux (1, 7). Approaches targeting cancer metabolism such as inhibition of glycolysis and restriction of glutamine availability have shown promising results in preclinical and clinical studies (1, 7).

In addition to direct targeting of metabolic enzymes or transporters to restrict nutrient availability, modulation of mitochondrial metabolism that occurs through the tricarboxylic acid (TCA) cycle and electron transport chain (ETC) has been proposed as a novel approach for anticancer drug development (3, 9). The catabolic processing of sugars, glutamine, fatty acids, and amino acids leads to the generation of reducing equivalents in the form of NADH and FADH_2_, which are subsequently oxidized. The electrons transferred during this process are shuttled down the ETC, a process that generates an electrochemical gradient across the inner mitochondrial membrane necessary for ATP production. In addition to the crucial role of energy production, the TCA cycle also provides intermediates for lipid and amino acid synthesis. The increased demand for energy production and anabolic building blocks observed in cancer cells makes selectively targeting the TCA cycle and ETC promising approaches to limiting malignancy and proliferation. It is noteworthy that the type II diabetes drug metformin has been reported to inhibit mitochondrial complex I activity and provide beneficial effects on a number of cancer models (10–12). A recent study indicates that metformin blocks gluconeogenesis resulting from the inhibition of mitochondrial glycerol-3-phosphate dehydrogenase, another contributor of electrons to ETC (13). This newly discovered activity of metformin could also be responsible in part for the reduction in risk of cancer and diminished cancer-related mortality in patients using the drug (12).

Lonidamine (LND; 1-(2,4-dichlorobenzyl)-1*H*-indazole-3-carboxylic acid) has been used in combination with other therapeutic agents to improve efficacy and overall response to cancer treatment (14–18). Following the initial clinical trials, recent progress in developing LND nanoparticle and liposome delivery systems represents potential new approaches for its clinical application (19–22). Although the mechanism of action remains unclear, LND treatment is known to target metabolic pathways in cancer cells. Early studies based on reduced secreted lactate claimed that glycolysis was inhibited in LND-treated cells (16, 23, 24). This was proposed to arise through LND-mediated inhibition of mitochondrially bound hexokinase II, the enzyme that catalyzes the phosphorylation of glucose to glucose 6-phosphate once it enters the cell (23). LND was subsequently shown to inhibit monocarboxylic acid transporters, which prevents lactate export from cells and causes intracellular acidification (25–27). It was also reported that LND inhibits mitochondrial ETC (28). However, the mechanism underlying this inhibition was not clearly delineated.

By utilizing liquid chromatography (LC)-selected reaction monitoring/mass spectrometry (MS) coupled with isotopologue analysis, we have determined that LND inhibits mitochondrial complex II, resulting in alterations in the TCA cycle and glutamine metabolism in the DB-1 melanoma cell line. Thus, we have established a previously unknown pharmacological activity of LND. This finding may hold promise for novel therapeutic combinations and provide new approaches for the treatment of cancer.

## Experimental Procedures

### 

#### 

##### Chemicals and Reagents

Rotenone, 4,4,4-trifluoro-1-(2-thienyl)-1,3-butanedione (TTFA), 3-nitropropionic acid (3-NPA), malonate, 2,6-dichlorophenolindophenol, decylubiquinone, 2-(4,5-dimethyl-2-thiazolyl)-3,5-diphenyl-2*H*-tetrazolium bromide (MTT), phenazine methosulfate (PMS), carbonyl cyanide 4-(trifluoromethoxy)phenylhydrazone (FCCP),[^13^C_5_,^15^N_1_]glutamate, methoxyamine HCl, diisopropylethylamine (DIPEA), pentafluorobenzyl bromide, 1,1,1,3,3,3-hexafluoro-2-propanol, dimethyl sulfoxide (DMSO), 2′,7′-dichlorofluorescin diacetate, *N*-acetylcysteine, propidium iodide (PI), succinate, [^13^C_4_]succinate, [^13^C_3_]lactate, [^13^C_6_]citrate, and [^13^C_5_,^15^N_2_]glutamine were purchased from Sigma-Aldrich. LND was purchased from Santa Cruz Biotechnology. Optima LC-MS grade water, methanol, acetonitrile, and isopropanol were purchased from Thermo Fisher Scientific (Waltham, MA). [^13^C_4_]Fumarate and glutathione ([^13^C_2_,^15^N_1_]glycine) were purchased from Cambridge Isotope Laboratories (Tewksbury, MA).

##### Cell Culture and Treatment

DB-1 cells were human melanoma cells derived from a lymph node metastasis as described previously (29). DB-1 and HepG2 were maintained in minimum Eagle's medium α and RPMI 1640 medium, respectively. HeLa and HCT116 were cultured in DMEM. All the media were supplemented with 10% fetal bovine serum, 100 units/ml penicillin, and 100 mg/liter streptomycin. LND, TTFA, and 3-NPA were freshly prepared in DMSO. DB-1 cells were grown to 80% confluence and treated for 1 h with vehicle (0.1% DMSO) or the indicated treatment in glutamine-free DMEM containing 5 mm glucose. HepG2, HeLa, and HCT116 were treated with DMSO or LND (150 μm) for 1 h in their respective media. Cell volumes were determined using a Multisizer 3 Coulter Counter (Beckman Coulter).

##### Mitochondria Labeling by [^13^C_4_]Succinate

Mouse liver mitochondria were freshly prepared from adult mice as described previously (30). 60 μg of isolated mitochondria were suspended in 200 μl of reaction buffer (135 mm sucrose, 65 mm KCl, 5 mm KH_2_PO_4,_ 10 mm Tris/HCl, 20 μm EGTA, 2.5 mm MgCl_2_, pH 7.4) containing 5 mm [^13^C_4_]succinate. Mitochondria samples were pulse-sonicated for 5 s before incubation at 37 °C for 30 min.

##### Organic Acid Extraction and Derivatization

Cells were washed twice with phosphate-buffered saline (PBS) followed by scraping into 750 μl of ice-cold methanol/water (4:1, v/v) containing 500 ng of internal standard ([^13^C_3_]lactate, [^13^C_4_]succinate, [^13^C_6_]citrate, and [^13^C_4_]fumarate). Samples were pulse-sonicated for 30 s and centrifuged at 16,000 × *g* for 10 min. The supernatant was then transferred to a glass tube. For α-ketoglutarate derivatization, methoxyamine HCl (2 mg) was added, and samples were incubated at 37 °C for 1 h. Following incubation, samples were evaporated to dryness under nitrogen and suspended in 100 μl of mobile phase A (400 mm 1,1,1,3,3,3-hexafluoro-2-propanol and 10 mm DIPEA in water) prior to LC-MS analysis. For NADPH and NADP^+^ analysis, the supernatant from methanol/water extracts was diluted 1:4 using 50 mm ammonium carbonate for LC-MS analysis.

For labeled mitochondria, 800 μl of ice-cold methanol containing 500 ng of [^13^C_5_,^15^N_1_]glutamate was added to quench the reaction. For quantifying glutamine in the culturing medium, 5 μl of medium were added to 500 μl of ice-cold methanol/water (4:1, v/v) containing 1.5 μg of [^13^C_5_,^15^N_2_]glutamine. Samples were pulse-sonicated for 30 s and centrifuged at 16,000 × *g* for 10 min. The supernatant was transferred to a clean tube and evaporated to dryness under nitrogen. The dried residues were derivatized with 100 μl of DIPEA in acetonitrile (0.5:99.5, v/v) and 100 μl of pentafluorobenzyl bromide in acetonitrile (1:4, v/v) at 60 °C for 1 h. Derivatized samples were evaporated to dryness under nitrogen and resuspended in hexanes/ethanol (95:5, v/v) prior to LC-electron capture atmospheric pressure chemical ionization-MS analysis (31).

##### LC-Selected Reaction Monitoring/MS Analysis

Organic acids from cell samples were analyzed using an Agilent 1200 series HPLC system coupled to an Agilent 6460 triple quadrupole mass spectrometer equipped with an electrospray ionization source operated in negative ion mode. Analytes were separated by reversed-phase ion-pairing chromatography utilizing a Phenomenex Luna C_18_ column (250 × 2.00 mm, 3-μm particle size) at a flow rate of 200 μl/min maintained at 45 °C. A two-solvent gradient system was used with solvent A as 400 mm 1,1,1,3,3,3-hexafluoro-2-propanol and 10 mm DIPEA in water and solvent B as 300 mm 1,1,1,3,3,3-hexafluoro-2-propanol and 10 mm DIPEA in methanol. The linear gradient conditions were as follows: 2% B at 0 min, 2% B at 3 min, 10% B at 28 min, 95% B at 31 min, 95% B at 38 min, 2% B at 39 min followed by a 6-min equilibration. The Agilent 6460 mass spectrometer operating conditions were as follows. The gas temperature was set at 320 °C, and the gas flow was set to 8 liters/min. The sheath gas temperature was 400 °C, and the sheath gas flow was set to 10 liters/min. The capillary voltage was set to 3000 V, and the nozzle voltage was set to 1000 V. The isotopic distribution of TCA cycle metabolites was calculated as described previously (32). The analysis of NADPH and NADP^+^ was similar except that an LC method with a 15-min run time was performed on a 150 × 2-mm Phenomenex Luna C_18_ column. This ensured that all the samples were analyzed within 3 h after the extraction and minimized decomposition of the NADPH and NADP^+^.

[^13^C_4_]Fumarate and [^13^C_4_]malate generated in mitochondria and glutamine from the medium were converted to pentafluorobenzyl bromide derivatives and analyzed as described previously (33). Briefly, pentafluorobenzyl bromide derivatives were separated using a CHIRALPAK AD-H column (250 × 4.6 mm, 5-μm particle size; Daicel Chemical Industries, Ltd., Tokyo, Japan) at a flow rate of 1 ml/min maintained at 30 °C. A postcolumn addition (0.75 ml/min) of methanol was used. Solvent A was hexanes, and solvent B was isopropanol/methanol (1:1, v/v). The linear gradient was as follows: 1% B for 3 min, 60% B at 25 min, 60% B at 29 min, 1% at 30 min followed by a 5-min equilibration. MS analysis was performed on a Thermo Quantum Triple Stage Quadrupole mass spectrometer (Thermo Scientific) with an atmospheric pressure chemical ionization source operated in the negative ion mode. The operating conditions were as follows: vaporizer temperature, 350 °C; heated capillary temperature, 300 °C; corona discharge needle, 30 μA. The sheath gas and auxiliary gas pressures were 35 and 10 (arbitrary units), respectively. Cellular glutathione (GSH) levels were quantified as described previously (34).

##### Mitochondrial Oxygen Consumption Assay

For measurements of oxygen consumption via complex II, 80 μg of isolated mouse liver mitochondria were added to respiration buffer (135 mm sucrose, 65 mm KCl, 5 mm KH_2_PO_4_, 10 mm Tris/HCl, 20 μm EGTA, 2.5 mm MgCl_2_, pH 7.4) containing 5 mm succinate and LND at the indicated concentrations. ADP (100 μm) and FCCP (60 nm) were added sequentially. The measurements were carried out using a Clark electrode (model 949, Strathkelvin Instruments) in a water-jacketed glass reaction vessel (Mitocell MT200) containing 100 μl of stirred, air-saturated respiration buffer at 37 °C.

##### Enzyme Assays

Succinate-ubiquinone reductase (SQR) activity and succinate dehydrogenase (SDH) activity were measured as described (35, 36) with minor modifications. Briefly, mouse liver mitochondria were resuspended in assay buffer (0.3 m mannitol, 25 mm KH_2_PO_4_, pH 7.4) at 20 μg/ml and supplemented with 20 mm succinate, 5 μm rotenone, 2 μm antimycin A, and 10 mm NaN_3_. Following a 10-min incubation at room temperature, inhibitors were added and incubated for an additional 15 min before initiation of reactions. For SQR activity, reactions were initiated by adding 50 μm decylubiquinone and 50 μm 2,6-dichlorophenolindophenol (extinction coefficient = 21 mm^−1^ cm^−1^). Absorbance at 600 nm was monitored every min for 20 min. For the SDH activity of complex II, reactions were initiated by adding 150 μm MTT and 400 μm PMS. The change in absorbance of MTT was monitored at 570 nm for 20 min.

##### Intracellular Reactive Oxygen Species (ROS) Measurement

Cells were treated with vehicle or drugs for 4 h followed by staining with 2′,7′-dichlorofluorescin diacetate (10 μm) in minimum Eagle's medium α for 20 min at 37 °C. Cells were then trypsinized and rinsed twice with PBS. The cellular fluorescence of the oxidized product, 2′,7′-dichlorofluorescein, was analyzed by flow cytometry (Accuri C6, BD Biosciences). At least 10,000 data events were collected for each sample.

##### Propidium Iodide Staining for Cell Viability

After treatment with vehicle or drugs for 24–48 h, cells were trypsinized and rinsed twice with PBS. Cells resuspended in PBS were mixed with PI (final concentration of 1 μg/ml) right before analysis by flow cytometry (Accuri C6). At least 10,000 data events were collected for each sample.

##### Dynamic Isotopic Labeling of Cells

DB-1 cells were treated with DMSO, LND, or TTFA for 2 h before culturing in glutamine-free DMEM containing 2 mm [^13^C_5_,^15^N_2_]glutamine supplemented with 10% fetal bovine serum and drugs. After 0, 0.5, 1, 2, 4, and 6 h of incubation, medium was aspirated, and 750 μl of ice-cold methanol/water (4:1, v/v) were added to cell culture plates snap frozen on dry ice. The glucose labeling experiment was performed similarly except that cells were labeled with glucose-free DMEM containing [^13^C_6_]glucose, 10% fetal bovine serum, and relevant drugs. Cells were harvested after 0, 1, 2, 5, 10, 30, and 60 min of incubation.

##### Metabolic Flux Analysis Using ^13^C Metabolic Fragmented Mass Isotopologue Distribution Modeling

A two-compartment (extracellular medium, intracellular cellular distribution) metabolic model was used to fit experimental ^13^C dynamic mass isotopologues of labeled citrate and malate to determine metabolic fluxes. The metabolic network included glycolysis, the TCA cycle, extracellular glutamine uptake, cellular glutamate production via cytosolic and mitochondrial glutaminase, reductive carboxylation of α-ketoglutarate, anaplerosis through pyruvate carboxylase, glutaminolysis and malic enzyme activity.

The model was expressed mathematically using two types of mass balance equations: 1) mass balance for total metabolite concentration and 2) ^13^C mass isotopologue mass balance for labeled metabolites and their related fragments based on bionetwork and atom distribution matrices (fragmented mass isotopologue framework) (37). Mass isotopologue dynamics of the system were formulated as the Cauchy initial value problem for ordinary differential equations using mass isotopologue fractions as the state variables. Mass isotopologue balance equations were derived in a similar manner as equations for bonded cumulative isotopologues as described previously (38). In terms of ordinary differential equations, this model describes the rates of loss and creation of particular labeled and unlabeled metabolite forms (mass isotopologues) after incubations with labeled glutamine in extracellular media. For the [^13^C_5_,^15^N_2_]glutamine experiment, the fitted experimental dynamic mass isotopologues were seven mass isotopologue forms of citrate (unlabeled, M + 1 to M + 6 mass isotopologues) and five forms of malate with a total of 12 dynamic mass isotopologue curves. The extracted absolute fluxes for glutaminolysis and reductive carboxylation were determined. For all metabolite mass isotopologues, the ^13^C natural abundance of ^13^C isotope (1.078%) was taken into account.

Solving a system of non-linear differential equations in terms of whole/fragmented mass isotopologues with the Runge-Kutta fourth order procedure for stiff systems provided time courses for all possible ^13^C mass isotopologues (*e.g.* citrate and glutamate). Dynamic mass isotopologue values at the experimental time points were taken into account for fitting procedures. The cost function was used to quantify differences between measurements and computational results for labeled dynamic data and to select the corresponding vector of fluxes that minimizes the cost function in [^13^C_5_,^15^N_2_]glutamine experiments. Minimization was performed with simplex or Broyden-Fletcher-Goldfarb-Shanno algorithms. Correct mean square convergence was confirmed by verifying that goodness of fit values were close to expected theoretical values. To overcome potential local minima, several sets of initial random fluxes were used (4).

Reliability of the flux values was evaluated by using Monte-Carlo simulations as described previously (39). The Monte-Carlo simulation procedure was performed by generation of synthetic dynamic mass isotopologues for citrate and malate by solving differential equations. Metabolic fluxes, which were representative for metabolic fluxes in DB-1 melanoma cells, were used as nominal values to generate these synthetic dynamic mass isotopologues. For each Monte-Carlo draw (at least 500 draws total), random Gaussian noise with a mean of zero and standard deviation σ of 0.01 was added to dynamic ^13^C mass isotopologues. The chosen noise level was slightly above a typical noise level for *in vitro* ^13^C LC-MS cell studies. These synthetic renormalized dynamic mass isotopologues were then fitted using a ^13^C metabolic model to obtain best fit values. Different starting values were chosen randomly to ensure that the results were independent of the initial condition of the fit. Resulting parametric probability density functions directly reflect the uncertainty of each fitted parameter. Distributions were characterized by their standard deviations. Other statistics not reported include probability density functions, confidence intervals, and cross-correlation between metabolic fluxes/parameters. All numerical procedures were carried out in Matlab (Mathworks, Natick, MA) as described previously (4).

## Results

### 

#### 

##### LND Treatment Results in the Accumulation of Succinate

**FIGURE 1.**
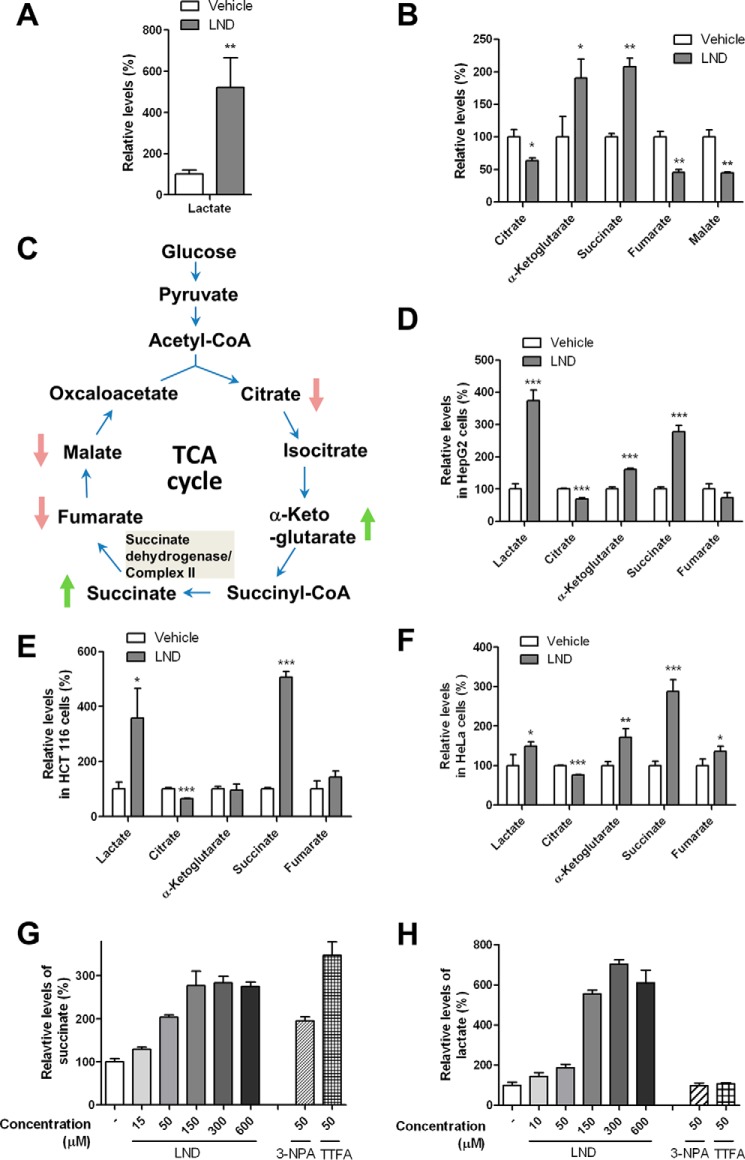
**LND treatment alters the levels of TCA cycle metabolites.** *A* and *B*, DB-1 cells were incubated with 150 μm LND or DMSO for 1 h. Levels of lactate (*A*) and metabolites in the TCA cycle (*B*) were measured by LC-MS. The levels of metabolites in LND-treated group were normalized with respect to the relevant metabolites in DMSO controls. *C*, a scheme of the TCA cycle. The changes observed in LND-treated cells are indicated. HepG2 (*D*), HCT116 (*E*), and HeLa (*F*) cells were incubated with DMSO or LND (150 μm) for 1 h. Levels of lactate and TCA metabolites were quantified by LC-MS. *G* and *H*, DB-1 cells were treated with DMSO (−), LND, 3-NPA, or TTFA at the indicated concentrations. Levels of succinate and lactate were quantified by LC-MS. For *A*, *B*, and *D–H*, the means of three samples are shown. *Error bars* represent S.D. *, *p* < 0.05; **, *p* < 0.01; ***, *p* < 0.001 (Student's *t* test).

To examine the effect of LND on melanoma cell metabolism, we used LC-selected reaction monitoring/MS to analyze the metabolites in DB-1 melanoma cells treated with LND. As a monocarboxylate transporter inhibitor, LND is known to stimulate lactate accumulation in cells (25, 26). After 1 h of treatment with 150 μm LND, lactate was elevated nearly 5-fold over control cells (Fig. 1*A*). The levels of succinate and α-ketoglutarate in LND-treated DB-1 cells were found to increase, whereas the levels of citrate, fumarate, and malate decreased (Fig. 1, *B* and *C*). A 3–5-fold accumulation of succinate was also observed in a variety of cancer cell lines, including HepG2 (Fig. 1*D*), HCT116 (Fig. 1*E*), and HeLa (Fig. 1*F*).

The accumulation of succinate and decrease in fumarate and malate in DB-1 cells suggested that the conversion of succinate to fumarate was inhibited by LND (Fig. 1*C*). The oxidation of succinate to fumarate is catalyzed by the succinate dehydrogenase activity of complex II. Therefore, we investigated the effect of LND on succinate accumulation in DB-1 cells at various concentrations and compared its potency with two known complex II inhibitors, 3-NPA and TTFA. At 50 μm, both LND and 3-NPA induced a 2-fold increase in succinate levels, whereas TTFA treatment resulted in a larger increase (Fig. 1*G*). Neither 3-NPA nor TTFA affected lactate levels (Fig. 1*H*). LND failed to induce further succinate or lactate accumulation in DB-1 cells beyond a dose of 150 μm (Fig. 1, *G* and *H*). This observation indicates that cells may not be able to effectively take up LND from the cell culture medium at concentrations beyond 150 μm.

##### LND Inhibits the Formation of Fumarate and Malate in Isolated Mitochondria

**FIGURE 2.**
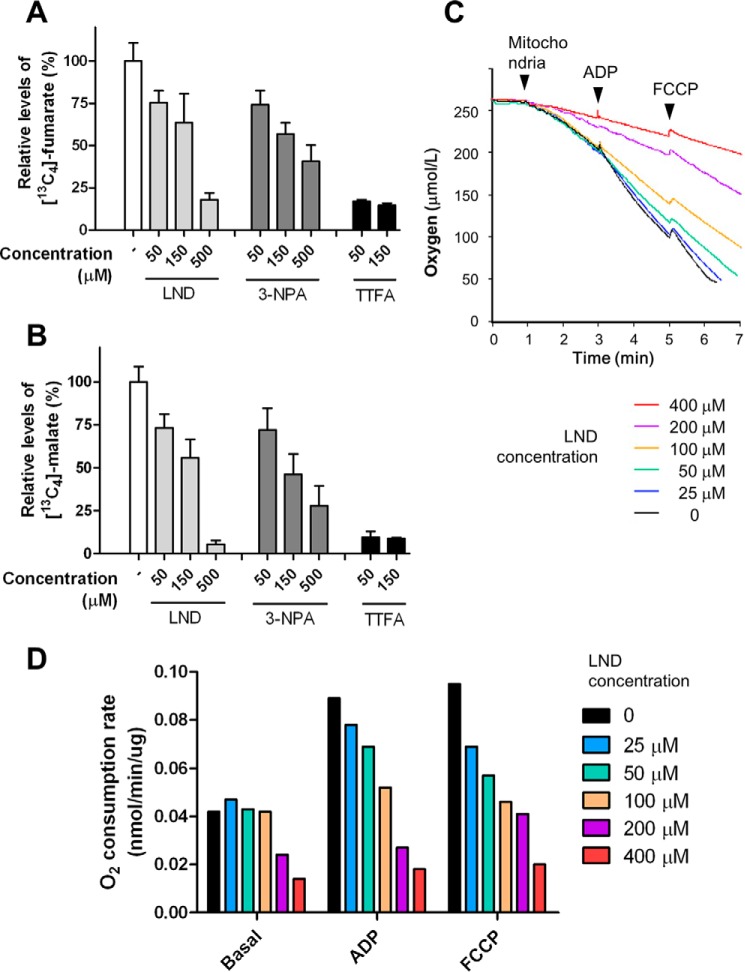
**LND inhibits complex II activity in isolated mitochondria.** *A* and *B*, isolated mouse liver mitochondria were incubated with DMSO (−) or the indicated drugs and 5 mm [^13^C_4_]succinate at 37 °C for 30 min. The levels of [^13^C_4_]fumarate (*A*) and [^13^C_4_]malate (*B*) generated in each treatment were normalized with respect to the relevant metabolites in the vehicle-treated group. The data presented are means of three samples. *Error bars* represent S.D. *C* and *D*, isolated mouse liver mitochondria were incubated with 5 mm succinate, 5 μm rotenone, and LND at the indicated concentrations. *C*, oxygen consumption was measured by a Clark electrode. *Arrowheads* indicate the addition of mitochondria, ADP, and FCCP. *D*, quantification of oxygen consumption rate in (*C*) expressed as nmol of oxygen/min/μg of mitochondrial protein.

To rule out the possibility that the inhibition of the succinate dehydrogenase activity of complex II by LND was an indirect effect resulting from metabolic alterations in the cytosol, we examined the conversion of succinate to fumarate using isolated mitochondria. Importantly, the utilization of isolated mitochondria reduces interference of cytoplasmic metabolites and enzymes. To interrogate the specific action of complex II, isolated mitochondria were incubated with 5 mm [^13^C_4_]succinate. The generation of [^13^C_4_]fumarate, [^13^C_4_]malate, and [^13^C_4_]citrate was quantified to assess succinate metabolism. Within 30 min of incubation, 106 nmol of [^13^C_4_]fumarate and 232 nmol of [^13^C_4_]malate were generated from 60 μg of mitochondria with vehicle treatment. No heavy labeled citrate was detected, most likely due to the lack of external acetyl-CoA supply. The addition of LND inhibited the production of [^13^C_4_]fumarate and [^13^C_4_]malate (Fig. 2, *A* and *B*). 150 μm LND inhibited ∼40% of fumarate and 50% of malate production. The extent of inhibition caused by LND was similar to that of 3-NPA at various concentrations, whereas TTFA was a more potent inhibitor of complex II (Fig. 2, *A* and *B*). Overall, our observations suggest that the inhibition of complex II activity by LND is independent of lactate accumulation or other metabolic changes in the cytosol.

##### LND Inhibits Complex II-dependent Respiration

Floridi and Lehninger (28) have previously explored the ability of LND to inhibit cellular respiration. To examine the effect of LND on complex II-dependent respiration, we included the complex I inhibitor rotenone in the respiration assay utilizing succinate as the metabolic substrate. We measured the mitochondrial oxygen consumption rate at the basal level, in the ADP-stimulated condition, and in the presence of the mitochondrial uncoupler FCCP (Fig. 2*C*). LND inhibited mitochondrial oxygen consumption rates under all three conditions. Similar to that observed in the previous study (28), the inhibition was much stronger under ADP- and FCCP-stimulated conditions than at basal levels (Fig. 2, *C* and *D*). LND did not inhibit the basal respiration up to 100 μm. Under all conditions, half-maximal inhibition by LND was observed between 100 and 200 μm.

##### Mechanism of Complex II Inhibition by LND

**FIGURE 3.**
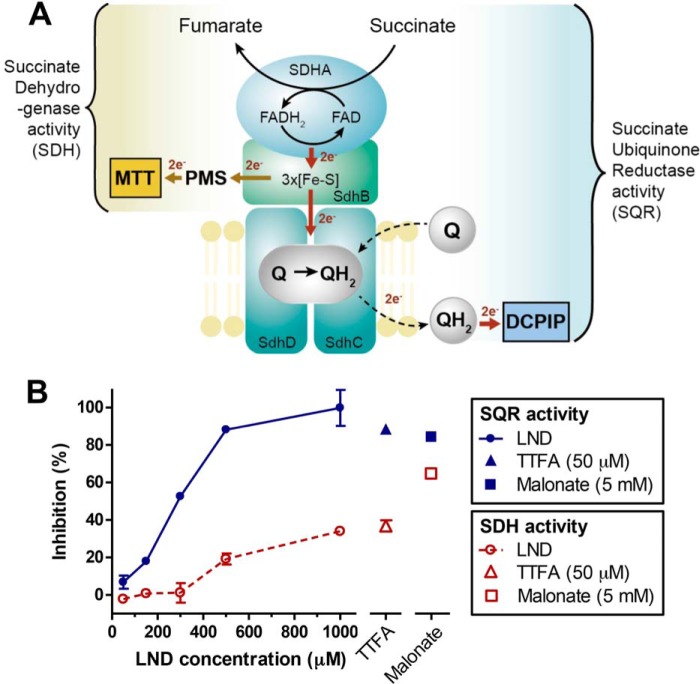
**LND inhibits the SQR activity of complex II.** *A*, a schematic representation of complex II subunits and enzyme activities. SQR activity was determined by measuring the electron transfer from succinate to decylubiquinone and 2,6-dichlorophenolindophenol (*DCPIP*). SDH of complex II was measured by the electron transfer from succinate to the iron-sulfur cluster and finally to the water-soluble dyes PMS and MTT. *Q*, ubiquinone; *QH_2_*, reduced ubiquinone. *B*, isolated mouse liver mitochondria were incubated with LND, TTFA, and malonate at the indicated concentrations. SQR and SDH activities were measured as described in *A*. The percentage of inhibition was determined by comparing the enzyme activities of the drug-treated groups with DMSO-treated controls. The data presented are means of three samples. *Error bars* represent S.D. The SQR and SDH activities of complex II in all treatment groups were determined on the same batch of freshly prepared mitochondria.

Complex II contains four subunits: A (flavoprotein), B (iron-sulfur subunit), C (15-kDa integral membrane protein), and D (cytochrome *b* small subunit) encoded by the *SDHA*, *SDHB*, *SDHC*, and *SDHD* genes, respectively. Electrons obtained from the oxidation of succinate within SDHA are transferred from the SDHA-bound flavin adenine dinucleotide (FAD) cofactor to the Fe-S clusters of SDHB and finally to the ubiquinone reduction site residing between SDHC and SDHD where ubiquinone is reduced to ubiquinol (Fig. 3*A*). To determine the specificity of complex II inhibition by LND, we measured the formation of ubiquinol by complex II (SQR activity) using a water-soluble ubiquinone analogue decylubiquinone in combination with the artificial electron acceptor 2,6-dichlorophenolindophenol. In the SQR activity assay, isolated mouse liver mitochondria were incubated with succinate and decylubiquinone together with rotenone and the complex III inhibitor antimycin A. Our results showed that LND inhibited the formation of ubiquinol by complex II in a dose-dependent manner (Fig. 3*B*). At 500 μm, around 90% of the SQR activity was inhibited by LND.

To further investigate the mechanism of complex II inhibition by LND, we also examined the SDH activity of complex II. SDH activity is the first step of the SQR reaction and can be determined by two artificial electron acceptors, PMS and MTT. PMS and MTT create a bypass around ubiquinone in SDHC and SDHD and directly accept electrons from iron-sulfur clusters in subunit B. The SDH activity was determined by monitoring the color change of MTT (Fig. 3*A*).

To determine the specificity of the SQR and SDH activity assays, we compared the inhibition by TTFA and malonate in isolated mitochondria. TTFA inhibits complex II activity by primarily binding to the ubiquinone-binding site, whereas malonate competes with succinate for the dehydrogenase-binding site on complex II. TTFA (50 μm) potently inhibited SQR activity (89%) but exhibited only modest inhibition of MTT reduction (36%) as shown in Fig. 3*B*. In contrast, malonate (5 mm) inhibited SQR by 84% and MTT reduction by 65%.

Increasing doses of LND were used to compare its effect on the SQR and SDH activities of complex II in isolated mitochondria. The inhibition of SQR activity was found to be much greater than inhibition of SDH activity at all LND concentrations tested. LND at 1 mm almost completely inhibited the activity of SQR, whereas the SDH activity was only reduced by 34% (Fig. 3*B*). This finding indicates that LND failed to fully block the electron transfer from the iron-sulfur cluster to PMS and MTT. Thus, LND appears to inhibit complex II activity by interfering with the ubiquinone reduction, possibly at the ubiquinone-binding site in SDHC and SDHD.

##### LND Induces ROS Formation and Cell Death

**FIGURE 4.**
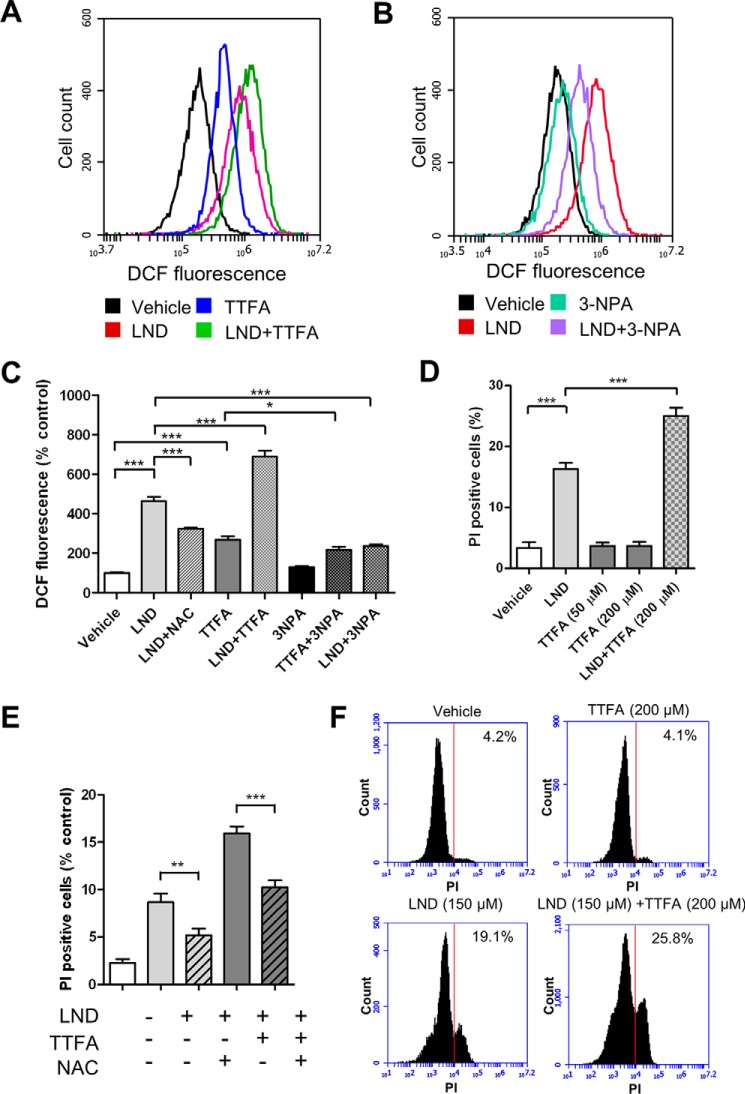
**LND induces cellular ROS release and reduces cell viability.** *A* and *B*, ROS in DB-1 cells was measured by 2′,7′-dichlorofluorescein (*DCF*) fluorescence. Shown are the representative flow cytometry histograms of samples treated with DMSO or the indicated drugs. *C*, mean fluorescence intensities of cells treated with DMSO or the indicated drugs. *D* and *E*, PI exclusion method was used to examine the viability of DB-1 cells treated with DMSO or indicated drugs. Shown are the percentages of PI-positive cells. The data presented are means of three samples. *Error bars* represent S.D. *, *p* < 0.05; **, *p* < 0.01; ***, *p* < 0.001 (Student's *t* test). *F*, representative flow cytometry histograms for PI-stained cells from *D*. Percentages of PI-positive cells are indicated. Cells were treated with the indicated drugs for 48 (*D* and *F*) or 24 h (*E*). The following drug concentrations were used: LND, 150 μm; TTFA, 150 μm unless indicated otherwise; 3-NPA, 500 μm; *N*-acetylcysteine (*NAC*), 10 mm.

As part of the ETC, complex II is also a source of ROS. It mainly produces ROS from either the reduced FAD or ubiquinone site when downstream components of the ETC are blocked (40, 41). To examine whether LND induces intracellular ROS generation, we quantified the level of ROS by 2′,7′-dichlorofluorescein fluorescence (Fig. 4, *A–C*). Surprisingly, the level of ROS formed in LND-treated cells was even higher than the level in cells treated with TTFA (Fig. 4, *A* and *C*).

To determine whether LND induces ROS through complex II inhibition, we examined ROS generation from a combined treatment with LND and TTFA or LND and 3-NPA. 3-NPA binds to the active site of SDHA and inhibits the electron transfer from succinate to FAD. Thus, 3-NPA alone does not trigger substantial ROS production and can reduce ROS induced by TTFA, which inhibits complex II at the ubiquinone-binding site (34, 36) (Fig. 4*C*). LND and TTFA treatments showed an additive effect of ROS production. In contrast, the addition of 3-NPA markedly decreased the level of ROS generated in LND-treated cells (Fig. 4, *A–C*). This result suggests that LND induces ROS at a site within complex II downstream of SDHA. It also supports our finding that LND inhibits complex II by interfering with ubiquinone reduction.

LND also induced substantial cell death after treatment of DB-1 cells for 48 (Fig. 4*D*) or 24 h (Fig. 4*E*). TTFA alone was unable to induce large amounts of cell death. However, it significantly promoted cell death triggered by LND (Fig. 4, *D* and *F*). The increased cell death caused by the addition of TTFA to LND-treated cells indicates that LND renders cells vulnerable to further complex II inhibition or additional ROS. Incubation with the ROS scavenger *N*-acetylcysteine reduced the rates of cell death in cells treated with LND alone or with TTFA (Fig. 4, *C* and *E*). Thus, ROS is responsible, at least in part, for cell death induction under these conditions.

##### LND Reduces Cellular Levels of Glutathione and NADPH and the PPP

**FIGURE 5.**
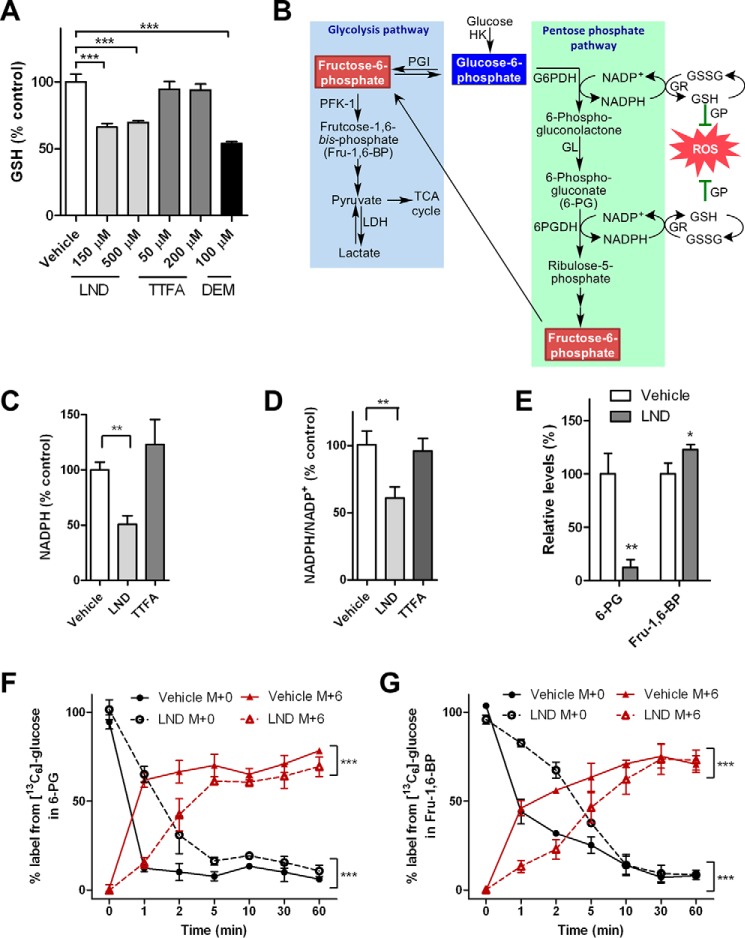
**Effect of LND on GSH, NADPH, and pentose phosphate pathway.** *A*, GSH levels of DB-1 cells treated with DMSO or the indicated drugs were quantified by LC-MS. *B*, a scheme showing the links among glycolysis, the PPP, NADPH production, and GSH redox cycling. *G6PDH*, glucose-6-phospate dehydrogenase; *6-PGDH*, 6-phosphogluconate dehydrogenase; *GL*, gluconolactonase; *GP*, glutathione peroxidase; *GR*, glutathione reductase; *GSSG*, glutathione disulfide; *LDH*, lactate dehydrogenase; *PFK-1*, phosphofructokinase 1; *PGI*, phosphoglucose isomerase; *HK*, hexokinase. *C* and *D*, cells treated with DMSO, LND (150 μm), or TTFA (150 μm) for 4 h. NADPH and NADP^+^ were measured by LC-MS. Shown are the relative levels of NADPH (*C*) and relative ratios of NADPH/NADP^+^ (*D*). *E*, DB-1 cells were incubated with DMSO or LND (150 μm) for 1 h. Levels of 6-phoshogluconate (*6-PG*) and fructose 1,6-bisphosphate (*Fru-1,6-BP*) were measured by LC-MS. *F* and *G*, DB-1 cells treated with DMSO or LND (150 μm) were labeled with [^13^C_3_]glucose for the indicated times. Shown are the ^13^C labeling percentages of 6-phosphogluconate (*F*) and fructose 1,6-bisphosphate (*G*) plotted over time. *p* values between the control and LND-treated groups were calculated by two-way analysis of variance with repeated measures. For *A* and *C–G*, the means of three samples are shown. *Error bars* represent S.D. *, *p* < 0.05; **, *p* < 0.01; ***, *p* < 0.001.

To investigate why the LND-treated cells were more susceptible to complex II inhibition than TTFA-treated cells, levels of the cellular antioxidant GSH were compared. LND caused a 40% drop in GSH levels at a concentration of 150 μm and above (Fig. 5*A*). The extent of GSH reduction is similar to that caused by 100 μm diethyl maleate (DEM), a compound known to deplete GSH (Fig. 5*A*). In contrast, TTFA (50 or 200 μm) caused a modest reduction of 6% (Fig. 5*A*). Consistent with the reduced GSH levels, the levels of NADPH and the NADPH/NADP^+^ ratio both decreased after LND treatment but not after TTFA treatment (Fig. 5, *C* and *D*). The PPP is an important source of NADPH (5) required for the GSH reductase-mediated reduction of glutathione disulfide back to GSH (42). LND has been reported previously to be an inhibitor of hexokinase (Fig. 5*B*) (23, 43), which catalyzes the first step of glycolysis. In contrast, some studies have shown no effect on hexokinase based on the analysis of glucose 6-phosphate after LND treatment of breast cancer and glioma cell lines (25, 26, 43). However, these latter studies did not examine the effect of LND on specific PPP metabolites, which could also be down-regulated as a result of hexokinase inhibition (Fig. 5*B*). In support of this possibility, we found that the concentration of 6-phosphogluconate, an important PPP metabolite, was markedly decreased in LND-treated DB-1 cells (Fig. 5*E*). In addition, a time course for the incorporation of [^13^C_6_]glucose into PPP metabolites (determined by LC-MS) revealed that incorporation into the M + 6 isotopologue [^13^C_6_]6-phosphogluconate as well as the glycolysis metabolite [^13^C_6_]fructose 1,6-bisphosphate was significantly delayed (Fig. 5, *F* and *G*). These data, together with the reduced levels of 6-phosphogluconate, suggest that the flux into the PPP was greatly reduced by LND, most likely through inhibition of hexokinase. Thus, the reduced NADPH levels (Fig. 5*C*) and NADPH/NADP^+^ ratios (Fig. 5*D*) and decreased GSH levels (Fig. 5*A*) in LND-treated cells resulted, in part, from inhibition of the PPP (Fig. 5*B*).

##### GSH Depletion Sensitizes Melanoma Cells to LND or TTFA treatment

**FIGURE 6.**
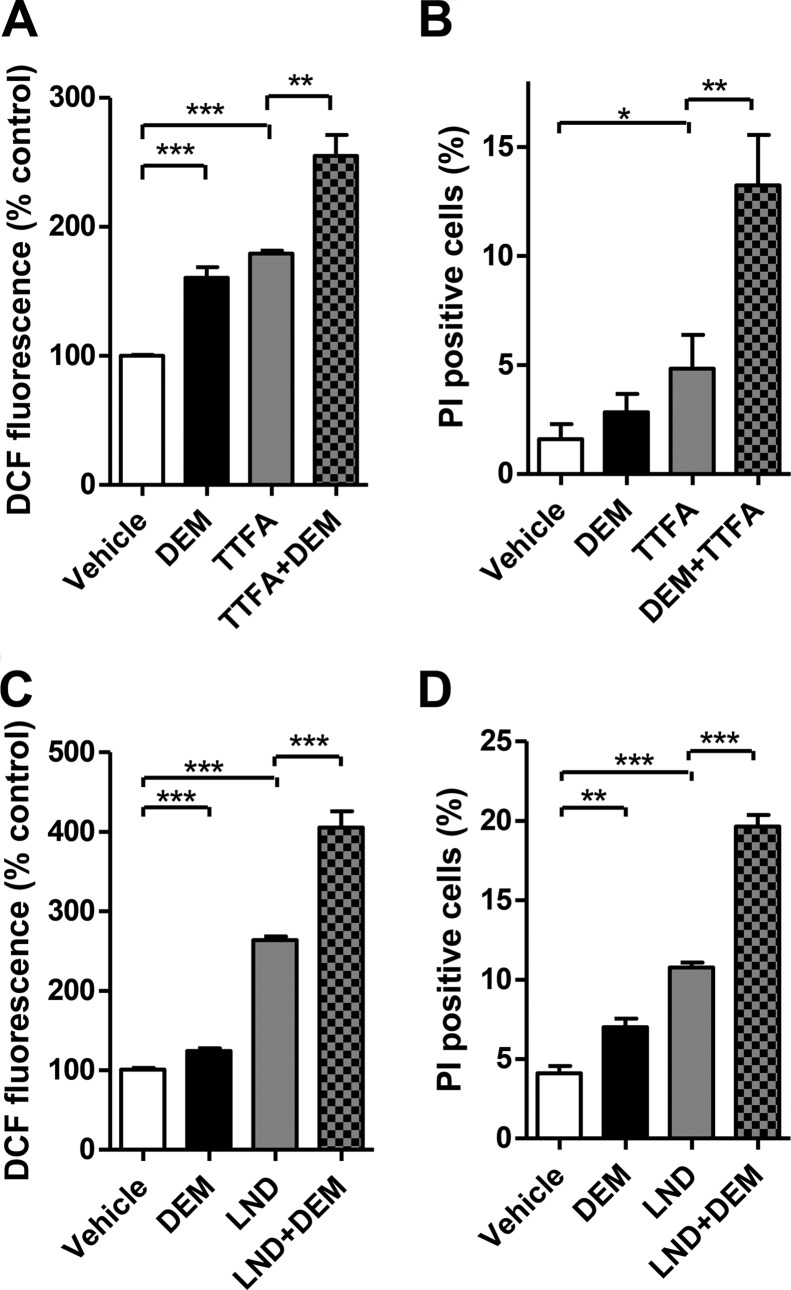
**GSH depletion enhances the ROS and cell death triggered by LND and TTFA.** 2′,7′-Dichlorofluorescein fluorescence (*DCF*) (*A* and *C*) and cell viability (*B* and *D*) of DB-1 cells treated with DMSO or the indicated drugs were measured as described in Fig. 4. 100 μm DEM was used for each treatment. The data presented are means of three samples. *Error bars* represent S.D. *, *p* < 0.05; **, *p* < 0.01; ***, *p* < 0.001 (Student's *t* test).

The reduced PPP and GSH levels caused by LND may render cells more susceptible to damage from ROS. To test this hypothesis, we incubated DB-1 cells with LND or TTFA in the presence or absence of the GSH depletion agent DEM. TTFA induced less ROS production (Fig. 6*A*) and less cell death (Fig. 6*B*) than LND (Fig. 6, *C* and *D*). However, DEM triggered more ROS in the TTFA-treated cells than TTFA alone (Fig. 6*A*). Noticeably, a combination of TTFA and DEM induced significantly more cell death compared with the rates in the individual drug treatments (Fig. 6*B*). Similar results were observed for the combined treatment with LND and DEM (Fig. 6, *C* and *D*), although the magnitudes of the responses were greater than what was observed with TTFA/DEM (Fig. 6, *A* and *B*).

##### LND and TTFA Increase the Contribution of Glutamine to the TCA cycle through Oxidative Metabolism in Melanoma Cells

**FIGURE 7.**
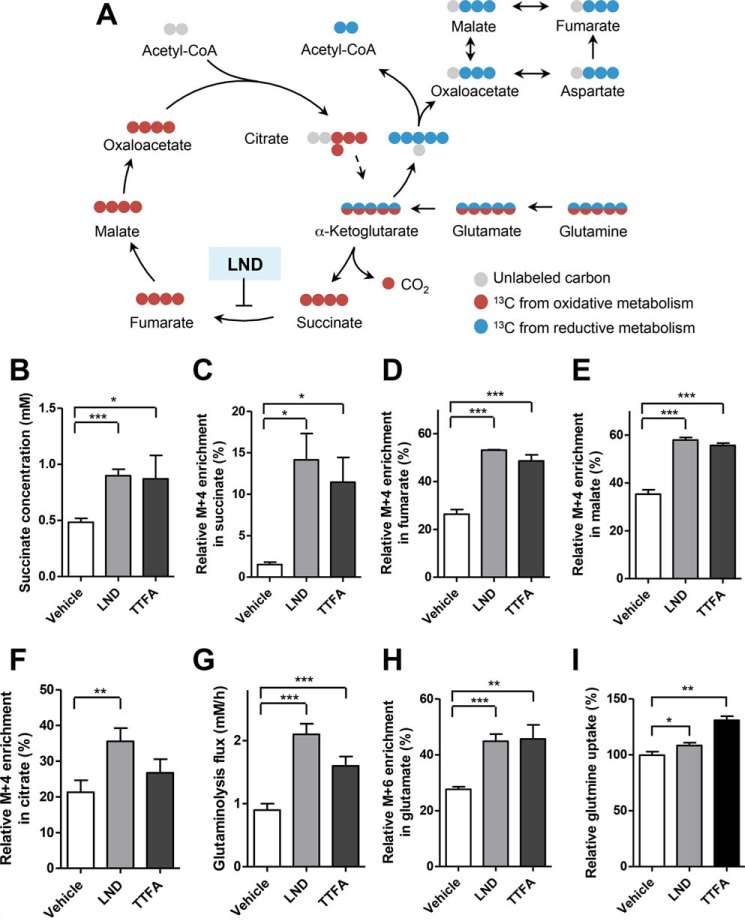
**LND and TTFA reduce oxidative glutamine metabolism in DB-1 cells.** *A*, a scheme showing the ^13^C labeling of metabolites by [^13^C_5_,^15^N_2_]glutamine in the oxidative and reductive glutamine metabolism. *B–H*, DB-1 cells were treated with DMSO, LND (300 μm), or TTFA (50 μm) for 2 h. Levels of succinate (*B*) and other metabolites (Table 1) were quantified in one set of samples, whereas the rest of the cells were further incubated with 2 mm [^13^C_5_,^15^N_2_]glutamine and the indicated drugs. After 6 h of incubation, the ^13^C labeling from the oxidative glutamine metabolism is shown by the M + 4 isotopic enrichment of succinate (*C*), fumarate (*D*), malate (*E*), and citrate (*F*). Rates of glutaminolysis as well as the standard deviation and *p* values were calculated as described under “Experimental Procedures” (*G*). The glutamate labeling is shown by M + 6 isotopic enrichment (*H*). *I*, glutamine uptake was measured by incubating cells with DMSO, LND (300 μm), or TTFA (50 μm) for 12 h. For all the panels, the means of three samples are shown. *Error bars* represent S.D. *, *p* < 0.05; **, *p* < 0.01; ***, *p* < 0.001.

**FIGURE 8.**
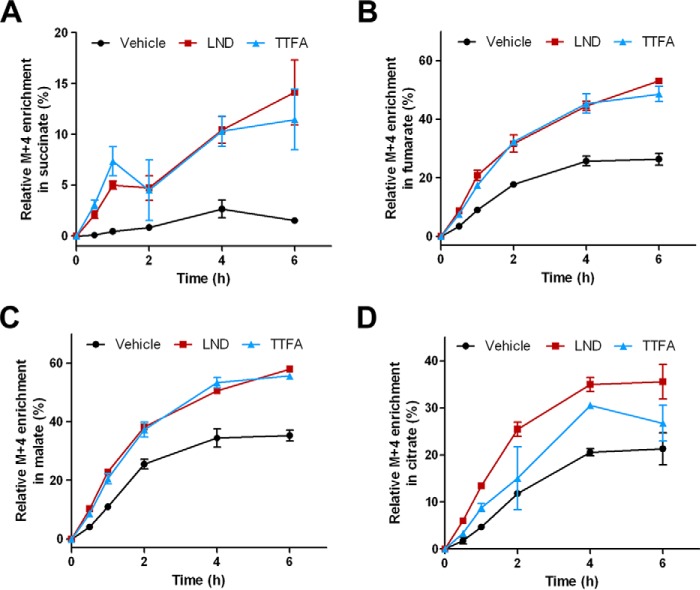
**Dynamic labeling through oxidative glutamine metabolism in LND- and TTFA-treated cells.** Dynamic labeling of DMSO-, LND- (150 μm), and TTFA (50 μm)-treated cells after switching to [^13^C_5_,^15^N_2_]glutamine. Data were collected from the same dynamic labeling experiment as described in Fig. 7. Labeling percentages of M + 4 succinate (*A*), fumarate (*B*), malate (*C*), and citrate (*D*) were plotted over time. The data presented are the means of three samples. *Error bars* represent S.D.

In addition to the effect on ROS generation and cell viability, we also further explored the metabolic changes triggered by LND and complex II inhibition. Glutamine is an important carbon source for TCA cycle anaplerosis in many cancer cells (Fig. 7*A*) (44). Therefore, we examined the effect of LND on glutamine metabolism and compared it with TTFA at a lower concentration so that they both induce a similar extent of succinate accumulation (Fig. 7*B*). Five carbon atoms from glutamine enter the TCA cycle via α-ketoglutarate followed by conversion to succinyl-CoA and succinate (Fig. 7*A*). Alternatively, α-ketoglutarate can be metabolized reductively to citrate to provide carbon atoms for lipid synthesis (Fig. 7*A*). We performed a dynamic labeling assay to analyze both oxidative and reductive pathways of glutamine metabolism. DB-1 cells were first treated with vehicle, LND, or TTFA and then cultured in medium containing [^13^C_5_,^15^N_2_]glutamine. Incorporation of [^13^C]carbons into TCA cycle metabolites at different time points was then determined. Over the 6 h of labeling, glutamine contributed to succinate, fumarate, malate, and citrate through oxidative metabolism as shown by their M + 4 isotopologues (Figs. 7 and 8). Most metabolites reached steady state labeling beyond 4 h in control cells (Fig. 8). Treatment of DB-1 cells with LND strikingly increased the labeling in succinate (Fig. 8*A*), fumarate (Fig. 8*B*), malate (Fig. 8*C*), and citrate (Fig. 8*D*) over the time course of the experiment as well as at steady state (Fig. 7, *C–F*). Similar effects were also observed in TTFA-treated cells (Figs. 7, *C–F*, and 8, *A–D*).

**FIGURE 9.**
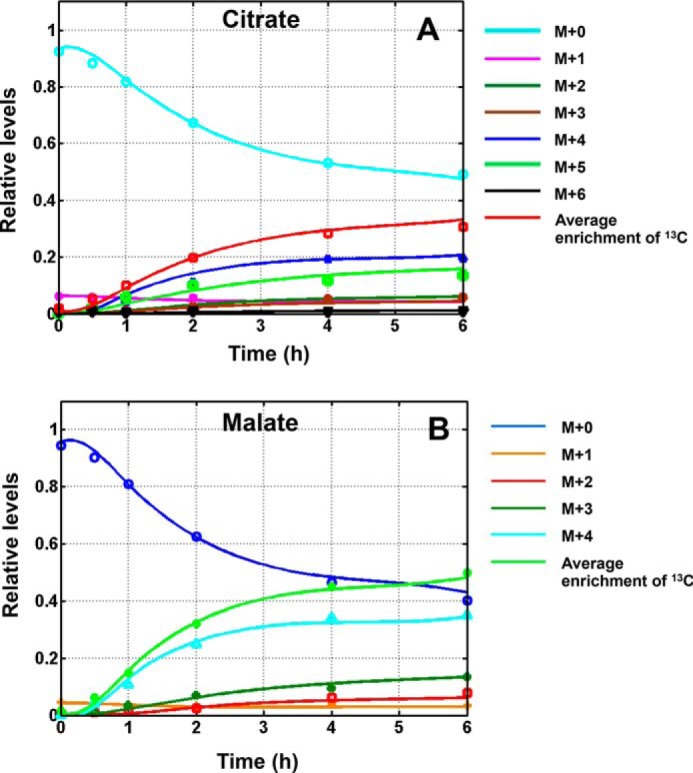
**Flux analysis of glutamine metabolism.** *A*, kinetics of citrate labeling in control cells after switching to [^13^C_5_,^15^N_2_]glutamine. *B*, kinetics of malate labeling in DMSO-treated cells after switching to [^13^C_5_,^15^N_2_]glutamine. *Solid lines* represent simulated labeling curves that best fit the data points.

To determine whether LND and TTFA altered flux from glutamine into TCA cycle metabolites, we integrated the kinetic labeling patterns with cellular concentrations of metabolites in a two-compartment metabolic flux model (Table 1 and Fig. 9). The analysis showed that glutaminolysis flux was significantly higher in LND- and TTFA-treated cells (Fig. 7*G*). Consistently, the M + 6 isotopologue of glutamate was also increased upon drug treatment (Fig. 7*H*), although its level remained unchanged (Table 1). The enhanced glutamine utilization in LND- and TTFA-treated cells is underscored by their increased glutamine uptake during a 12-h incubation (Fig. 7*I*). Although the increase in overall glutamine uptake is less than 10% for LND, this percentage was calculated based on the number of cells at the beginning of treatment. Given the arrested cell growth and cell death induced by LND, the actual glutamine uptake may be even higher.

**TABLE 1:** **Concentrations of metabolites quantified after 2 h of drug treatment (means ± S.D.; n = 3)**

	Mean ± S.D. (*n* = 3)		
	Vehicle	LND	TTFA
	*mm*		
Succinate	0.48 ± 0.03	0.90 ± 0.06	0.87 ± 0.2
Fumarate	0.35 ± 0.05	0.35 ± 0.05	0.28 ± 0.023
Malate	0.74 ± 0.08	0.96 ± 0.04	0.71 ± 0.03
Citrate	0.89 ± 0.004	0.27 ± 0.01	0.90 ± 0.06
Glutamate	28.08 ± 2.02	31.81 ± 2.03	27.01 ± 1.45

##### Glutamine-dependent Reductive Carboxylation Is Reduced by LND

**FIGURE 10.**
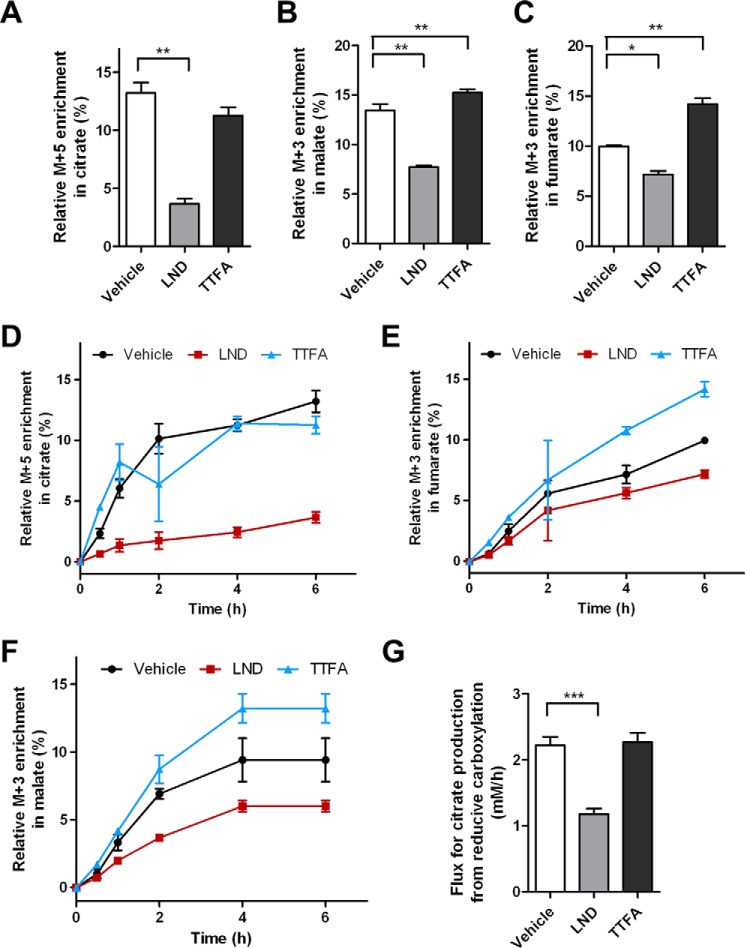
**Dynamic labeling through reductive glutamine metabolism in LND- and TTFA-treated cells.** Data were collected from the same labeling experiment as described in Figs. 7–9. After 6 h of incubation with [^13^C_5_,^15^N_2_]glutamine, metabolism by the reductive carboxylation pathway is shown for the M + 5 of citrate (*A*), M + 3 of malate (*B*), and M + 3 of fumarate (*C*). Labeling percentages of M + 5 citrate (*D*), M + 3 fumarate (*E*), and M + 3 malate (*F*) were plotted over time. The rates of reductive carboxylation to citrate were determined by flux analysis (*G*). The data presented are the means of three samples. *Error bars* represent S.D. *, *p* < 0.05; **, *p* < 0.01; ***, *p* < 0.001.

DB-1 cells also use glutamine through reductive metabolism (Fig. 7*A*) as demonstrated by the formation of malate and fumarate M + 3 isotopologues and the citrate M + 5 isotopologue (Fig. 10, *A–F*). LND, but not TTFA, reduced the labeling of these metabolites resulting from reductive glutamine metabolism over the dynamic labeling or at the steady state (Fig. 10, *A–F*). LND lowered the flux from reductive carboxylation into citrate by nearly 50%. In contrast, the reductive flux was unchanged in TTFA-treated cells (Fig. 10*G*).

## Discussion

LND is known to interfere with energy metabolism in cancer cells. To improve its efficacy and potentially reduce organ toxicity, recent studies have focused on developing targeted delivery systems for LND (19–22). For example, EGF receptor-targeting nanoparticles have significantly improved the tumor-suppressive effect of LND in combination with the chemotherapeutic agent paclitaxel (20). Utilizing delivery with the targeting nanocarriers, 25 nmol of LND/g of tissue was reached in tumor xenografts (19). It is important to note that the local cellular concentration of LND may be even higher, indicating that the doses used in this study may be physiologically relevant in these settings. Another nanoparticle delivery system that targets LND to mitochondria showed an increase of more than 100-fold in the efficacy of reducing cancer cell viability compared with non-targeted LND (21), further highlighting the importance of elucidating unknown mechanisms by which LND exerts antitumor effects.

The effect of LND on central energy metabolism has never been fully characterized. Floridi *et al.* (23, 28) reported that LND inhibits the respiration in both Ehrlich ascites cells and isolated mitochondria. However, the sites of inhibition were not clearly identified. Through LC-MS analysis of TCA cycle metabolites, we found that LND inhibited the oxidation of succinate to fumarate in various cell lines and in isolated mitochondria (Figs. 1 and 2). Furthermore, we showed that in the presence of artificial electron acceptors LND inhibited ubiquinone reduction, but it did not fully block the transfer of electrons from iron-sulfur clusters in SDHB (Fig. 3). These results indicate that SDH activity is not the target of LND. This notion is further supported by assays for ROS generation upon LND treatment (Fig. 4, *A–C*). TTFA and 3-NPA had additive and inhibitory effects on LND-triggered ROS, respectively, suggesting that free electrons are released from a location between the SDH active site and ubiquinone-binding site. Because LND effectively inhibits the SQR activity of complex II, we propose that LND inhibits complex II by interfering with the reduction of ubiquinone. However, it is unclear whether the inhibition is caused by direct blockade of the ubiquinone-binding site or through allosteric modifications to the tertiary structure of SDHC and SDHD.

Incubation of the DB-1 cells with the classical complex II inhibitor TTFA triggered less ROS release compared with that caused by LND treatment (Fig. 4, *A* and *C*). There was a concomitant increase in the amount of cell death in the LND-treated cells when compared with the TTFA-treated cells (Fig. 4, *D* and *F*). The increase in ROS production and cell death was amplified by DEM for both TTFA (Fig. 6, *A* and *C*) and LND treatments (Fig. 6, *B* and *D*). Importantly, TTFA induced a significant amount of additional cell death when combined with LND treatment (Fig. 4, *D* and *F*). These results suggest that the compromised antioxidant system in the LND-treated cells resulting from PPP inhibition (reduced NADPH and GSH) contributes to the susceptibility to cell death in response to complex II inhibition and ROS release.

Inhibition of TCA cycle enzymes can have consequences for tumor progression depending upon tumor types and the stage of tumorigenesis. For example, fumarate hydratase deficiency and complex II subunit mutations are both associated with the development of cancer (41, 45). Accumulation of succinate resulting from complex II deficiency inhibits prolyl hydroxylases in the cytosol, leading to stabilization and activation of HIF1α (46). Complex II inhibition can also induce ROS production (41, 47). Both of these pathways are involved in tumorigenesis (46–48). Our findings raise a concern that complex II inhibition by LND may be advantageous to certain tumor types. In line with this notion, both LND- and TTFA-treated cells showed increased labeling through oxidative glutamine metabolism (Fig. 7, *C–F*) as well as higher rates of glutaminolysis (Fig. 7*G*) and glutamine uptake (Fig. 7*I*). These effect are reminiscent of the greater reliance on glutamine by many cancer cells (44). Consistent with our findings, previous studies have shown that complex II knockdown or fumarate hydratase deficiency resulted in higher glutamine contribution to the TCA cycle and higher overall glutamine consumption (45, 49). It is still unknown why deficiencies in TCA cycle enzymes would result in a higher consumption of glutamine. Nevertheless, our finding suggests that a combination of LND with a glutaminase inhibitor (50) could have a positive synergistic effect when used as an anticancer therapeutic approach.

Flux analysis was conducted to more fully understand the effect of LND treatment on the metabolism of DB-1 cells (Figs. 7*G* and 10*G*). This revealed that LND lowered the flux from reductive carboxylation of glutamine-derived α-ketoglutarate into citrate (Fig. 7*A*) by nearly 50% (Fig. 10*G*). In contrast, flux through the reductive carboxylation pathway was unchanged in TTFA-treated cells (Fig. 10*G*). Unlike the increase of oxidative glutamine metabolism, which is an effect of complex II inhibition (Figs. 7 and 8), no apparent changes in reductive metabolism were detected in TTFA-treated cells (Fig. 10). Thus, the effect on reductive glutamine metabolism was specific to LND when compared with a classical complex II inhibitor. Reductive carboxylation of glutamine-derived α-ketoglutarate is regulated by NADPH-dependent isocitrate dehydrogenase (51). Therefore, the LND-mediated reduction in GSH (Fig. 5*A*) and NADPH levels (Fig. 5*C*) and decreased NADPH/NADP^+^ ratio (Fig. 5*D*) are consistent with a decrease in the PPP (Fig. 5*B*) and decreased flux through the reductive carboxylation of glutamine-derived α-ketoglutarate to citrate (Fig. 10*G*).

Interestingly, the utility of complex II inhibitors as anticancer drugs has been explored despite the potential risk for increased tumorigenesis (52). Several ubiquinone-binding site inhibitors such as α-tocopheryl succinate and its analogues have shown promising results in inducing apoptosis of cancer cells and suppressing tumor growth (52–56). In agreement with our findings, it has been shown that ROS generated upon complex II inhibition can induce apoptosis of cancer cells (36, 57, 58). In our study, LND effectively induced cell death of DB-1 melanoma cells due, in part, to its ability to induce ROS production through complex II inhibition (Fig. 4). At the same time, LND also diminished the capacity of cellular antioxidant systems by suppressing the PPP-derived NADPH production and subsequent GSH regeneration (Fig. 5). Many proposed pro-oxidant cancer therapies are based on either increasing cellular ROS or reducing cellular antioxidant defense systems (59, 60). These two combined activities of LND could make it an effective therapeutic option as a synergistic anticancer drug. In fact, the effectiveness of LND in combination with doxorubicin, melphalan, or radiotherapy for the treatment of breast, brain, melanoma, prostate, and ovarian tumors has already been tested (17, 18, 22, 61–63).

## Author Contributions

I. A. B., J. D. G., and D. B. L. conceived and coordinated the study. L. G., A. J. W., and K. N. performed the experiments. D. S. N. and A. A. S. performed the flux analysis. I. A. B, L. G., A. J. W., and J. D. G. analyzed the data and wrote the paper. All authors reviewed the results and approved the final version of the manuscript.

## Footnotes

3 The abbreviations used are:

PPP: pentose phosphate pathway
3-NPA: 3-nitropropionic acid
CHC: α-cyano-4-hydroxycinnamate
DEM: diethyl maleate
DIPEA: diisopropylethylamine
DMSO: dimethyl sulfoxide
ETC: electron transport chain
FCCP: carbonyl cyanide 4-(trifluoromethoxy)phenylhydrazone
GSH: glutathione
LND: lonidamine
MTT: 2-(4,5-dimethyl-2-thiazolyl)-3,5-diphenyl-2*H*-tetrazolium bromide
PI: propidium iodide
PMS: phenazine methosulfate
ROS: reactive oxygen species
SDH: succinate dehydrogenase
SQR: succinate-ubiquinone reductase
TCA: tricarboxylic acid
TTFA: 4,4,4-trifluoro-1-(2-thienyl)-1,3-butanedione.

## References

[1] Galluzzi L., Kepp O., Vander Heiden M. G., and Kroemer G. (2013) Metabolic targets for cancer therapy. Nat. Rev. Drug Discov. 12, 829–846 24113830 10.1038/nrd4145

[2] Granchi C., Fancelli D., and Minutolo F. (2014) An update on therapeutic opportunities offered by cancer glycolytic metabolism. Bioorg. Med. Chem. Lett. 24, 4915–4925 25288186 10.1016/j.bmcl.2014.09.041

[3] Weinberg S. E., and Chandel N. S. (2015) Targeting mitochondria metabolism for cancer therapy. Nat. Chem. Biol. 11, 9–15 25517383 10.1038/nchembio.1712 PMC4340667

[4] Mehrmohamadi M., Liu X., Shestov A. A., and Locasale J. W. (2014) Characterization of the usage of the serine metabolic network in human cancer. Cell Rep. 9, 1507–1519 25456139 10.1016/j.celrep.2014.10.026 PMC4317399

[5] Patra K. C., and Hay N. (2014) The pentose phosphate pathway and cancer. Trends Biochem. Sci. 39, 347–354 25037503 10.1016/j.tibs.2014.06.005 PMC4329227

[6] Sotgia F., Martinez-Outschoorn U. E., and Lisanti M. P. (2013) Cancer metabolism: new validated targets for drug discovery. Oncotarget 4, 1309–1316 23896568 10.18632/oncotarget.1182 PMC3787159

[7] Vander Heiden M. G. (2011) Targeting cancer metabolism: a therapeutic window opens. Nat. Rev. Drug Discov. 10, 671–684 21878982 10.1038/nrd3504

[8] Warburg O., Posener K., and Negelein E. (1924) Ueber den stoffwechsel der tumoren. Biochem. Z. 152, 319–344

[9] Fulda S., Galluzzi L., and Kroemer G. (2010) Targeting mitochondria for cancer therapy. Nat. Rev. Drug Discov. 9, 447–464 20467424 10.1038/nrd3137

[10] Evans J. M., Donnelly L. A., Emslie-Smith A. M., Alessi D. R., and Morris A. D. (2005) Metformin and reduced risk of cancer in diabetic patients. BMJ 330, 1304–1305 15849206 10.1136/bmj.38415.708634.F7 PMC558205

[11] Wheaton W. W., Weinberg S. E., Hamanaka R. B., Soberanes S., Sullivan L. B., Anso E., Glasauer A., Dufour E., Mutlu G. M., Budigner G. S., and Chandel N. S. (2014) Metformin inhibits mitochondrial complex I of cancer cells to reduce tumorigenesis. Elife 3, e02242 24843020 10.7554/eLife.02242 PMC4017650

[12] Franciosi M., Lucisano G., Lapice E., Strippoli G. F., Pellegrini F., and Nicolucci A. (2013) Metformin therapy and risk of cancer in patients with type 2 diabetes: systematic review. PLoS One 8, e71583 23936520 10.1371/journal.pone.0071583 PMC3732236

[13] Madiraju A. K., Erion D. M., Rahimi Y., Zhang X. M., Braddock D. T., Albright R. A., Prigaro B. J., Wood J. L., Bhanot S., MacDonald M. J., Jurczak M. J., Camporez J. P., Lee H. Y., Cline G. W., Samuel V. T., Kibbey R. G., and Shulman G. I. (2014) Metformin suppresses gluconeogenesis by inhibiting mitochondrial glycerophosphate dehydrogenase. Nature 510, 542–546 24847880 10.1038/nature13270 PMC4074244

[14] De Lena M., Lorusso V., Latorre A., Fanizza G., Gargano G., Caporusso L., Guida M., Catino A., Crucitta E., Sambiasi D., and Mazzei A. (2001) Paclitaxel, cisplatin and lonidamine in advanced ovarian cancer. A phase II study. Eur. J. Cancer 37, 364–368 11239758 10.1016/s0959-8049(00)00400-7

[15] Di Cosimo S., Ferretti G., Papaldo P., Carlini P., Fabi A., and Cognetti F. (2003) Lonidamine: efficacy and safety in clinical trials for the treatment of solid tumors. Drugs Today 39, 157–174 12730701 10.1358/dot.2003.39.3.799451

[16] Floridi A., Bruno T., Miccadei S., Fanciulli M., Federico A., and Paggi M. G. (1998) Enhancement of doxorubicin content by the antitumor drug lonidamine in resistant Ehrlich ascites tumor cells through modulation of energy metabolism. Biochem. Pharmacol. 56, 841–849 9774146 10.1016/s0006-2952(98)00054-9

[17] Nath K., Nelson D. S., Heitjan D. F., Leeper D. B., Zhou R., and Glickson J. D. (2015) Lonidamine induces intracellular tumor acidification and ATP depletion in breast, prostate and ovarian cancer xenografts and potentiates response to doxorubicin. NMR Biomed. 28, 281–290 25504852 10.1002/nbm.3240 PMC4361034

[18] Nath K., Nelson D. S., Ho A. M., Lee S. C., Darpolor M. M., Pickup S., Zhou R., Heitjan D. F., Leeper D. B., and Glickson J. D. (2013) ^31^P and ^1^H MRS of DB-1 melanoma xenografts: lonidamine selectively decreases tumor intracellular pH and energy status and sensitizes tumors to melphalan. NMR Biomed. 26, 98–105 22745015 10.1002/nbm.2824 PMC3465621

[19] Milane L., Duan Z. F., and Amiji M. (2011) Pharmacokinetics and biodistribution of lonidamine/paclitaxel loaded, EGFR-targeted nanoparticles in an orthotopic animal model of multi-drug resistant breast cancer. Nanomedicine 7, 435–444 21220050 10.1016/j.nano.2010.12.009 PMC3136558

[20] Milane L., Duan Z., and Amiji M. (2011) Therapeutic efficacy and safety of paclitaxel/lonidamine loaded EGFR-targeted nanoparticles for the treatment of multi-drug resistant cancer. PLoS One 6, e24075 21931642 10.1371/journal.pone.0024075 PMC3169576

[21] Marrache S., and Dhar S. (2012) Engineering of blended nanoparticle platform for delivery of mitochondria-acting therapeutics. Proc. Natl. Acad. Sci. U.S.A. 109, 16288–16293 22991470 10.1073/pnas.1210096109 PMC3479596

[22] Li N., Zhang C. X., Wang X. X., Zhang L., Ma X., Zhou J., Ju R. J., Li X. Y., Zhao W. Y., and Lu W. L. (2013) Development of targeting lonidamine liposomes that circumvent drug-resistant cancer by acting on mitochondrial signaling pathways. Biomaterials 34, 3366–3380 23410681 10.1016/j.biomaterials.2013.01.055

[23] Floridi A., Paggi M. G., D'Atri S., De Martino C., Marcante M. L., Silvestrini B., and Caputo A. (1981) Effect of lonidamine on the energy metabolism of Ehrlich ascites tumor cells. Cancer Res. 41, 4661–4666 7306982

[24] Natali P. G., Salsano F., Viora M., Nista A., Malorni W., Marolla A., and De Martino C. (1984) Inhibition of aerobic glycolysis in normal and neoplastic lymphoid cells induced by lonidamine [1-(2,4-dichlorobenzyl)-I-H-indazol-3-carboxylic acid]. Oncology 41, Suppl. 1, 7–14 6609324 10.1159/000225879

[25] Ben-Yoseph O., Lyons J. C., Song C. W., and Ross B. D. (1998) Mechanism of action of lonidamine in the 9L brain tumor model involves inhibition of lactate efflux and intracellular acidification. J. Neurooncol. 36, 149–157 9525814 10.1023/a:1005819604858

[26] Ben-Horin H., Tassini M., Vivi A., Navon G., and Kaplan O. (1995) Mechanism of action of the antineoplastic drug lonidamine: ^31^P and ^13^C nuclear magnetic resonance studies. Cancer Res. 55, 2814–2821 7796408

[27] Fang J., Quinones Q. J., Holman T. L., Morowitz M. J., Wang Q., Zhao H., Sivo F., Maris J. M., and Wahl M. L. (2006) The H^+^-linked monocarboxylate transporter (MCT1/SLC16A1): a potential therapeutic target for high-risk neuroblastoma. Mol. Pharmacol. 70, 2108–2115 17000864 10.1124/mol.106.026245

[28] Floridi A., and Lehninger A. L. (1983) Action of the antitumor and antispermatogenic agent lonidamine on electron transport in Ehrlich ascites tumor mitochondria. Arch. Biochem. Biophys. 226, 73–83 6227286 10.1016/0003-9861(83)90272-2

[29] Hill L. L., Korngold R., Jaworsky C., Murphy G., McCue P., and Berd D. (1991) Growth and metastasis of fresh human melanoma tissue in mice with severe combined immunodeficiency. Cancer Res. 51, 4937–4941 1893383

[30] Frezza C., Cipolat S., and Scorrano L. (2007) Organelle isolation: functional mitochondria from mouse liver, muscle and cultured fibroblasts. Nat. Protoc. 2, 287–295 17406588 10.1038/nprot.2006.478

[31] Singh G., Gutierrez A., Xu K., and Blair I. A. (2000) Liquid chromatography/electron capture atmospheric pressure chemical ionization/mass spectrometry: analysis of pentafluorobenzyl derivatives of biomolecules and drugs in the attomole range. Anal. Chem. 72, 3007–3013 10939360 10.1021/ac000374a

[32] Fernandez C. A., Des Rosiers C., Previs S. F., David F., and Brunengraber H. (1996) Correction of ^13^C mass isotopomer distributions for natural stable isotope abundance. J. Mass Spectrom. 31, 255–262 8799277 10.1002/(SICI)1096-9888(199603)31:3<255::AID-JMS290>3.0.CO;2-3

[33] Worth A. J., Basu S. S., Snyder N. W., Mesaros C., and Blair I. A. (2014) Inhibition of neuronal cell mitochondrial complex I with rotenone increases lipid β-oxidation, supporting acetyl-coenzyme A levels. J. Biol. Chem. 289, 26895–26903 25122772 10.1074/jbc.M114.591354 PMC4175330

[34] Zhu P., Oe T., and Blair I. A. (2008) Determination of cellular redox status by stable isotope dilution liquid chromatography/mass spectrometry analysis of glutathione and glutathione disulfide. Rapid Commun. Mass Spectrom. 22, 432–440 18215009 10.1002/rcm.3380

[35] Miyadera H., Shiomi K., Ui H., Yamaguchi Y., Masuma R., Tomoda H., Miyoshi H., Osanai A., Kita K., and Omura S. (2003) Atpenins, potent and specific inhibitors of mitochondrial complex II (succinate-ubiquinone oxidoreductase). Proc. Natl. Acad. Sci. U.S.A. 100, 473–477 12515859 10.1073/pnas.0237315100 PMC141019

[36] Lemarie A., Huc L., Pazarentzos E., Mahul-Mellier A. L., and Grimm S. (2011) Specific disintegration of complex II succinate:ubiquinone oxidoreductase links pH changes to oxidative stress for apoptosis induction. Cell Death Differ. 18, 338–349 20706275 10.1038/cdd.2010.93 PMC3044456

[37] Muzykantov V. S., and Shestov A. A. (1986) Kinetic equations for the redistribution of isotopic molecules due to reversible dissociation. React. Kinet. Catal. Lett. 32, 307–312

[38] Shestov A. A., Valette J., Deelchand D. K., Uğurbil K., and Henry P. G. (2012) Metabolic modeling of dynamic brain ^13^C NMR multiplet data: concepts and simulations with a two-compartment neuronal-glial model. Neurochem. Res. 37, 2388–2401 22528840 10.1007/s11064-012-0782-5 PMC4806787

[39] Shestov A. A., Valette J., Uğurbil K., and Henry P. G. (2007) On the reliability of ^13^C metabolic modeling with two-compartment neuronal-glial models. J. Neurosci. Res. 85, 3294–3303 17393498 10.1002/jnr.21269

[40] Yankovskaya V., Horsefield R., Törnroth S., Luna-Chavez C., Miyoshi H., Léger C., Byrne B., Cecchini G., and Iwata S. (2003) Architecture of succinate dehydrogenase and reactive oxygen species generation. Science 299, 700–704 12560550 10.1126/science.1079605

[41] Dröse S. (2013) Differential effects of complex II on mitochondrial ROS production and their relation to cardioprotective pre- and postconditioning. Biochim. Biophys. Acta 1827, 578–587 23333272 10.1016/j.bbabio.2013.01.004

[42] Blair I. A. (2006) Endogenous glutathione adducts. Curr. Drug Metab. 7, 853–872 17168687 10.2174/138920006779010601

[43] Floridi A., Paggi M. G., Marcante M. L., Silvestrini B., Caputo A., and De Martino C. (1981) Lonidamine, a selective inhibitor of aerobic glycolysis of murine tumor cells. J. Natl. Cancer Inst. 66, 497–499 6937706

[44] Daye D., and Wellen K. E. (2012) Metabolic reprogramming in cancer: unraveling the role of glutamine in tumorigenesis. Semin. Cell Dev. Biol. 23, 362–369 22349059 10.1016/j.semcdb.2012.02.002

[45] Frezza C., Zheng L., Folger O., Rajagopalan K. N., MacKenzie E. D., Jerby L., Micaroni M., Chaneton B., Adam J., Hedley A., Kalna G., Tomlinson I. P., Pollard P. J., Watson D. G., Deberardinis R. J., Shlomi T., Ruppin E., and Gottlieb E. (2011) Haem oxygenase is synthetically lethal with the tumour suppressor fumarate hydratase. Nature 477, 225–228 21849978 10.1038/nature10363

[46] Selak M. A., Armour S. M., MacKenzie E. D., Boulahbel H., Watson D. G., Mansfield K. D., Pan Y., Simon M. C., Thompson C. B., and Gottlieb E. (2005) Succinate links TCA cycle dysfunction to oncogenesis by inhibiting HIF-α prolyl hydroxylase. Cancer Cell 7, 77–85 15652751 10.1016/j.ccr.2004.11.022

[47] Guzy R. D., Sharma B., Bell E., Chandel N. S., and Schumacker P. T. (2008) Loss of the SdhB, but not the SdhA, subunit of complex II triggers reactive oxygen species-dependent hypoxia-inducible factor activation and tumorigenesis. Mol. Cell. Biol. 28, 718–731 17967865 10.1128/MCB.01338-07 PMC2223429

[48] Rasola A., Neckers L., and Picard D. (2014) Mitochondrial oxidative phosphorylation TRAP(1)ped in tumor cells. Trends Cell Biol. 24, 455–463 24731398 10.1016/j.tcb.2014.03.005 PMC7670877

[49] Aspuria P. J., Lunt S. Y., Väremo L., Vergnes L., Gozo M., Beach J. A., Salumbides B., Reue K., Wiedemeyer W. R., Nielsen J., Karlan B. Y., and Orsulic S. (2014) Succinate dehydrogenase inhibition leads to epithelial-mesenchymal transition and reprogrammed carbon metabolism. Cancer Metab. 2, 21 25671108 10.1186/2049-3002-2-21 PMC4322794

[50] Emadi A., Jun S. A., Tsukamoto T., Fathi A. T., Minden M. D., and Dang C. V. (2014) Inhibition of glutaminase selectively suppresses the growth of primary acute myeloid leukemia cells with IDH mutations. Exp. Hematol. 42, 247–251 24333121 10.1016/j.exphem.2013.12.001

[51] Metallo C. M., Gameiro P. A., Bell E. L., Mattaini K. R., Yang J., Hiller K., Jewell C. M., Johnson Z. R., Irvine D. J., Guarente L., Kelleher J. K., Vander Heiden M. G., Iliopoulos O., and Stephanopoulos G. (2012) Reductive glutamine metabolism by IDH1 mediates lipogenesis under hypoxia. Nature 481, 380–384 22101433 10.1038/nature10602 PMC3710581

[52] Kluckova K., Bezawork-Geleta A., Rohlena J., Dong L., and Neuzil J. (2013) Mitochondrial complex II, a novel target for anti-cancer agents. Biochim. Biophys. Acta 1827, 552–564 23142170 10.1016/j.bbabio.2012.10.015

[53] Dong L. F., Low P., Dyason J. C., Wang X. F., Prochazka L., Witting P. K., Freeman R., Swettenham E., Valis K., Liu J., Zobalova R., Turanek J., Spitz D. R., Domann F. E., Scheffler I. E., Ralph S. J., and Neuzil J. (2008) α-Tocopheryl succinate induces apoptosis by targeting ubiquinone-binding sites in mitochondrial respiratory complex II. Oncogene 27, 4324–4335 18372923 10.1038/onc.2008.69 PMC2668987

[54] Neuzil J., Weber T., Schröder A., Lu M., Ostermann G., Gellert N., Mayne G. C., Olejnicka B., Nègre-Salvayre A., Stícha M., Coffey R. J., and Weber C. (2001) Induction of cancer cell apoptosis by α-tocopheryl succinate: molecular pathways and structural requirements. FASEB J. 15, 403–415 11156956 10.1096/fj.00-0251com

[55] Dong L. F., Jameson V. J., Tilly D., Cerny J., Mahdavian E., Marín-Hernández A., Hernández-Esquivel L., Rodríguez-Enríquez S., Stursa J., Witting P. K., Stantic B., Rohlena J., Truksa J., Kluckova K., Dyason J. C., Ledvina M., Salvatore B. A., Moreno-Sánchez R., Coster M. J., Ralph S. J., Smith R. A., and Neuzil J. (2011) Mitochondrial targeting of vitamin E succinate enhances its pro-apoptotic and anti-cancer activity via mitochondrial complex II. J. Biol. Chem. 286, 3717–3728 21059645 10.1074/jbc.M110.186643 PMC3030374

[56] Dong L. F., Freeman R., Liu J., Zobalova R., Marin-Hernandez A., Stantic M., Rohlena J., Valis K., Rodriguez-Enriquez S., Butcher B., Goodwin J., Brunk U. T., Witting P. K., Moreno-Sanchez R., Scheffler I. E., Ralph S. J., and Neuzil J. (2009) Suppression of tumor growth *in vivo* by the mitocan α-tocopheryl succinate requires respiratory complex II. Clin. Cancer Res. 15, 1593–1600 19223492 10.1158/1078-0432.CCR-08-2439

[57] Albayrak T., Scherhammer V., Schoenfeld N., Braziulis E., Mund T., Bauer M. K., Scheffler I. E., and Grimm S. (2003) The tumor suppressor cybL, a component of the respiratory chain, mediates apoptosis induction. Mol. Biol. Cell 14, 3082–3096 12925748 10.1091/mbc.E02-10-0631 PMC181552

[58] Quinlan C. L., Orr A. L., Perevoshchikova I. V., Treberg J. R., Ackrell B. A., and Brand M. D. (2012) Mitochondrial complex II can generate reactive oxygen species at high rates in both the forward and reverse reactions. J. Biol. Chem. 287, 27255–27264 22689576 10.1074/jbc.M112.374629 PMC3411067

[59] Wang J., and Yi J. (2008) Cancer cell killing via ROS: to increase or decrease, that is the question. Cancer Biol. Ther. 7, 1875–1884 18981733 10.4161/cbt.7.12.7067

[60] Ivanova D., Bakalova R., Lazarova D., Gadjeva V., and Zhelev Z. (2013) The impact of reactive oxygen species on anticancer therapeutic strategies. Adv. Clin. Exp. Med. 22, 899–908 24431321

[61] Amadori D., Frassineti G. L., De Matteis A., Mustacchi G., Santoro A., Cariello S., Ferrari M., Nascimben O., Nanni O., Lombardi A., Scarpi E., and Zoli W. (1998) Modulating effect of lonidamine on response to doxorubicin in metastatic breast cancer patients: results from a multicenter prospective randomized trial. Breast Cancer Res. Treat. 49, 209–217 9776504 10.1023/a:1006063412726

[62] Prabhakara S., and Kalia V. K. (2008) Optimizing radiotherapy of brain tumours by a combination of temozolomide and lonidamine. Indian J. Med. Res. 128, 140–148 19001677

[63] Kalia V. K., Prabhakara S., and Narayanan V. (2009) Modulation of cellular radiation responses by 2-deoxy-D-glucose and other glycolytic inhibitors: implications for cancer therapy. J. Cancer Res. Ther. 5, Suppl. 1, S57–S60 20009297 10.4103/0973-1482.55145

